# Spontaneous Enterococcus faecalis Meningitis in an Elderly Patient: A Rare Cause of Community-Acquired Bacterial Meningitis

**DOI:** 10.7759/cureus.119029

**Published:** 2026-11-02

**Authors:** João Loja, Rafael Ferreira Nascimento, João Miguel Freitas, Margarida Ornelas, Teresa Faria

**Affiliations:** 1 Internal Medicine, Serviço de Saúde da Região Autónoma da Madeira, Entidade Pública Empresarial da Região Autónoma da Madeira (SESARAM, EPERAM), Funchal, PRT; 2 Internal Medicine, Hospital Central do Funchal, SESARAM, EPERAM, Funchal, PRT

**Keywords:** cerebrospinal fluid (csf), elderly, enterococcus faecalis, lumbar puncture (lp), meningitis

## Abstract

*Enterococcus faecalis* *(E. faecalis)* is an uncommon cause of bacterial meningitis in adults, typically associated with neurosurgical procedures, central nervous system devices, or underlying comorbidities. Community-acquired meningitis caused by this microorganism is particularly rare. We report the case of a 76-year-old man who presented to the emergency department with acute prostration and altered mental status, without classical meningeal signs. Initial cranial CT revealed no acute abnormalities. Cerebrospinal fluid (CSF) analysis showed findings strongly suggestive of bacterial meningitis, and *E. faecalis* was subsequently identified as the causative pathogen. Intravenous ampicillin and ceftriaxone were administered, with progressive neurological improvement. An extensive diagnostic workup, including cranial MRI, transthoracic echocardiography, thoracoabdominal CT, and colonoscopy, failed to identify a definitive source of infection. This case report highlights the diagnostic challenges of *E. faecalis* meningitis in older adults, particularly in the absence of classical meningeal manifestations and an identifiable infectious source. It also underscores the importance of early recognition, comprehensive microbiological investigation, and appropriate antimicrobial therapy in the management of this rare clinical entity.

## Introduction

*Enterococcus faecalis (E. faecalis)* is a Gram-positive bacterium commonly found in the human gastrointestinal tract and an important cause of healthcare-associated infections [1]. Although it frequently causes urinary tract infections, bacteremia, and infective endocarditis, its role in central nervous system infections is uncommon [2]. Enterococcal meningitis accounts for approximately 0.3%-4% of reported bacterial meningitis cases, underscoring its rarity [2].

Most cases of *E. faecalis* meningitis occur in patients with predisposing factors, particularly a history of neurosurgery, cranial trauma, or the presence of intracranial devices [3]. Structural abnormalities of the skull base and cerebrospinal fluid (CSF) leaks may also facilitate bacterial invasion of the central nervous system [4,5]. Spontaneous enterococcal meningitis is generally associated with an infectious focus elsewhere in the body and typically occurs in patients with underlying comorbidities, such as malignancy, diabetes mellitus, or chronic kidney disease, as well as in individuals receiving immunosuppressive therapy [6].

We report the case of a 70-year-old patient with diabetes mellitus who presented with altered mental status and was subsequently diagnosed with *E. faecalis* meningitis. Despite an extensive diagnostic investigation, no definitive infectious source was identified. This case report highlights the importance of considering uncommon bacterial pathogens in patients presenting with unexplained altered mental status, even in the absence of typical neurosurgical or traumatic predisposing factors, and emphasizes the diagnostic challenges associated with spontaneous enterococcal meningitis.

## Case presentation

A 76-year-old man was brought to the emergency department due to acute-onset prostration and altered mental status. According to his family, the patient had been found at home by his wife earlier that morning, exhibiting a marked reduction in responsiveness and verbal communication. He had previously been independent in his activities of daily living, with no known history of cognitive impairment. The possibility of an accidental fall or medication overdose could not be excluded at the time of admission. His medical history was significant for arterial hypertension, type 2 diabetes mellitus, dyslipidemia, benign prostatic hyperplasia, chronic obstructive pulmonary disease, obesity, and chronic kidney disease. His regular medications included empagliflozin/metformin for diabetes mellitus. No known drug allergies were reported.

On initial examination, the patient was markedly obtunded, responding to tactile stimuli but unable to communicate verbally. His vital signs revealed a blood pressure of 122/70 mmHg, a heart rate of 76 beats per minute, and a body temperature of 37.0 °C. Pulmonary auscultation revealed crackles at the right lung base. The neurological examination was unremarkable, revealing no neck stiffness or other signs of meningeal irritation, with negative Kernig's and Brudzinski's signs. No other significant abnormalities were documented on the initial physical examination. Initial hemogram and biochemistry results are summarized in Table 1.

**Table 1:** Initial hemogram and biochemistry

Parameter	Result	Reference range
White blood cell count	4.4 x 10^9^/L	4.0-10.0 x 10^9^/L
Absolute neutrophil count	2.8 x 10^9^/L	1.9-7.2 x 10^9^/L
C-reactive protein	211 mg/dl	< 5 mg/dl
Hemoglobin	8.8 g/dl	13.7-17.3 g/dl
Platelet count	150 x 10^3^/uL	145-440 x 10^3^/uL
Serum creatinine	1.6 mg/dl	0.7-1.2 mg/dl
Serum potassium	4.2 mEq/L	3.5-5 mEq/L
Serum sodium	139 mEq/L	135-145 mEq/L
Serum glucose	226 mg/dl	82-115 mg/dl
Aspartate aminotransferase	10 U/L	< 40 U/L
Alanine aminotransferase	6.5 U/L	< 40 U/L

Initial laboratory investigations revealed microcytic hypochromic anemia and elevated inflammatory markers. No significant abnormalities in blood glucose or serum electrolyte levels were identified (Table 2). Arterial blood gas analysis showed no evidence of hypoxemia, hypercapnia, or acid-base disturbances that could account for the patient's altered mental status. A cranial CT scan showed no acute intracranial abnormalities. Given the persistence of altered mental status without an identifiable cause, a lumbar puncture was performed under sedation, yielding turbid CSF, which was subsequently sent for cytochemical and microbiological analysis. CSF molecular analysis and blood cultures were also collected, and the patient was hospitalized for further investigation and management. CSF analysis revealed marked pleocytosis, predominantly consisting of polymorphonuclear leukocytes, with a glucose concentration of 133 mg/dL and markedly elevated protein concentrations (Table 2).

**Table 2:** Cerebrospinal fluid (CSF) biochemical and cellular analysis, bacterial culture, and Gram staining

Parameter	Result	Reference range
Leucocyte count	5090 cells/mm³	0-5 cells/mm³
Leucocyte predominance	Neutrophilic	
Red blood cell count	113 cells/mm³	0-5 cells/mm³
Glucose	133 mg/dl	40-70 mg/dl
Protein	364 mg/dl	15-45 mg/dl
Gram stain	Numerous Gram-positive cocci	
Bacterial culture	Enterococcus faecalis	

Concurrent serum glucose was 226 mg/dL, yielding a CSF-to-serum glucose ratio of 0.59, suggesting that the elevated CSF glucose level was related to systemic hyperglycemia in the context of diabetes mellitus. Despite the absence of hypoglycorrhachia, the marked neutrophilic pleocytosis and elevated protein levels were suggestive of acute bacterial meningitis, prompting the initiation of empirical intravenous antimicrobial therapy with ceftriaxone and ampicillin. Molecular testing of the CSF for *Streptococcus pneumoniae, Neisseria meningitidis, Haemophilus influenzae, *and *Listeria monocytogenes* yielded negative results. CSF molecular testing using a multiplex meningitis/encephalitis panel yielded negative results.

However, the panel did not include *E. faecalis* among its target pathogens. Microbiological analysis of the CSF revealed numerous Gram-positive cocci on Gram staining. Subsequent aerobic CSF culture yielded *E. faecalis*, confirming the microbiological diagnosis of enterococcal meningitis (Table 2). Antimicrobial susceptibility testing demonstrated susceptibility to ampicillin and high-level gentamicin susceptibility, indicating the potential for synergistic activity with cell wall-active antibiotics. The combination of ampicillin and ceftriaxone was maintained due to the patient's underlying renal dysfunction, aiming to avoid the potential nephrotoxicity associated with aminoglycoside therapy. Blood cultures were negative.

As part of the diagnostic workup to identify a potential source of infection, contrast-enhanced CT of the chest, abdomen, and pelvis was performed. Thoracic and abdominal imaging were unremarkable. Additionally, diverticulosis of the descending and sigmoid colon was observed, along with mild wall thickening of the cecum and ascending colon, potentially suggestive of mild colitis. No other significant abnormalities or definitive infectious source were identified. Brain MRI revealed no acute intracranial abnormalities, focal parenchymal lesions, or diffusion restriction. No abnormal parenchymal or meningeal enhancement was identified. Transthoracic echocardiography was subsequently performed and revealed no significant abnormalities, with no echocardiographic evidence suggestive of infective endocarditis.

The patient completed a 21-day course of intravenous antibiotic therapy with ampicillin and ceftriaxone, achieving an excellent clinical response, with substantial neurological recovery and normalization of inflammatory markers. Following a favorable clinical course, he was discharged from the hospital and referred for outpatient follow-up in the Infectious Diseases Department.

## Discussion

Enterococcal meningitis is an infrequent but potentially life-threatening central nervous system infection [7]. In a comprehensive review of 140 cases of enterococcal meningitis, 59% occurred following neurosurgical procedures, whereas 41% were classified as spontaneous infections [3]. *E. faecalis *was responsible for approximately 76% of isolates identified at the species level, with an overall mortality rate of 21%, reaching 33% among patients with spontaneous meningitis [3]. These findings emphasize the clinical significance of this uncommon condition, particularly in patients with underlying comorbidities.

Spontaneous enterococcal meningitis has been associated with several predisposing conditions, including diabetes mellitus, malignancy, renal insufficiency, and immunosuppression [2]. Unlike postoperative infections, which are frequently related to direct bacterial inoculation or intracranial devices, spontaneous cases may result from hematogenous dissemination originating from a distant infectious focus [8]. In the present case, advanced age and type 2 diabetes mellitus were identified as potential predisposing factors, although their contribution to the development of infection cannot be definitively established. The patient had no known chronic kidney disease, no history of malignancy, and no known immunosuppressive conditions. An extensive etiological investigation, including thoracoabdominal CT, transthoracic echocardiography, and colonoscopy, did not identify a definitive infectious source. Although abdominal imaging revealed diverticulosis and mild colonic wall thickening, these findings were insufficient to establish a gastrointestinal origin.

The clinical presentation of bacterial meningitis in older adults may differ from that observed in younger individuals. In a prospective study involving 635 episodes of acute bacterial meningitis, Domingo et al. found that older patients were more likely to present with impaired consciousness and less likely to exhibit neck stiffness than younger adults [9]. Similarly, in a separate cohort of patients aged 80 years or older, the classical triad of fever, neck stiffness, and altered mental status was documented in only 43% of cases [10]. The present case highlights the importance of considering bacterial meningitis in older adults presenting with acute altered mental status, even in the absence of classical meningeal signs.

The combination of ampicillin and ceftriaxone has demonstrated clinical efficacy in *E. faecalis* infective endocarditis and is associated with a lower risk of nephrotoxicity than ampicillin plus gentamicin [11]. However, evidence supporting this dual beta-lactam regimen specifically for enterococcal meningitis remains limited. A published report of postoperative *E. faecalis* ventriculitis described microbiological clearance following treatment with ampicillin and ceftriaxone, suggesting that this combination may represent a therapeutic option in selected circumstances [12]. Nevertheless, differences in antimicrobial penetration into the central nervous system and the limited available clinical evidence prevent direct extrapolation of findings from infective endocarditis to enterococcal meningitis. Therefore, the favorable response observed in our patient should not be interpreted as establishing the efficacy or equivalence of this regimen compared with other therapeutic approaches.

Our patient completed a 21-day course of intravenous ampicillin and ceftriaxone, with substantial clinical improvement and normalization of inflammatory markers. Renal function was monitored throughout antimicrobial treatment, and no treatment-related adverse events were observed. The patient was subsequently discharged with outpatient Infectious Diseases follow-up. Although the clinical outcome was favorable, the optimal antimicrobial regimen and treatment duration for spontaneous enterococcal meningitis remain uncertain due to the limited available evidence. This case report highlights the importance of early recognition, microbiologically guided treatment, and careful clinical monitoring in the management of this rare infection.

## Conclusions

Although uncommon, *E. faecalis* meningitis is a potentially life-threatening infection requiring prompt diagnosis and appropriate antimicrobial treatment. This case report highlights the diagnostic challenges associated with bacterial meningitis in older adults, particularly when presenting with altered mental status in the absence of classical meningeal signs. Furthermore, the lack of an identifiable infectious source despite extensive investigation emphasizes the complexity of spontaneous enterococcal meningitis. The diagnosis was established through CSF analysis and microbiological investigation, highlighting the importance of integrating conventional culture methods with molecular diagnostic testing when evaluating suspected central nervous system infections. Early recognition, accurate pathogen identification, and timely initiation of targeted antimicrobial therapy are essential to improving clinical outcomes.
